# Seizure as an Initial Presentation for Posterior Reversible Encephalopathy Syndrome in Undiagnosed Systemic Lupus Erythematosus and Lupus Nephritis: A Case Report

**DOI:** 10.7759/cureus.10195

**Published:** 2020-09-02

**Authors:** Ethan N Hartman, Kubra Tuna, Elio Monsour, Karthikram Komanduri, Aneta Tarasiuk-Rusek

**Affiliations:** 1 Internal Medicine, University of Central Florida College of Medicine, Orlando, USA; 2 Internal Medicine, University of Central Florida College of Medicine, Ocala, USA; 3 Internal Medicine, University of Central Florida College of Medicine - Ocala Regional Medical Center, Ocala, USA; 4 Infectious Disease, University of Central Florida College of Medicine, Ocala, USA

**Keywords:** seizure, systemic lupus erythematosus, sle, posterior reversible encephalopathy syndrome, pres, lupus nephritis, hypertension

## Abstract

Posterior reversible encephalopathy syndrome (PRES) is a syndrome presenting with neurological manifestations including headaches, seizures, and notable changes in brain imaging. It is typically associated with an acute increase in blood pressure, metabolic abnormalities, and/or medication effects. PRES is challenging to diagnose due to its variable presentation and low incidence. Herein we describe a compelling case of PRES syndrome secondary to uncontrolled hypertension in the setting of systemic lupus erythematosus (SLE) and lupus nephritis.

## Introduction

Posterior reversible encephalopathy syndrome (PRES) is a clinical syndrome that presents with various neurological manifestations, including seizure, headache, and visual changes [1-11]. Computed tomography (CT) may visualize subtle areas of hypodensity, but magnetic resonance imaging (MRI) is considered the gold standard for the diagnosis of PRES [8]. The majority of PRES cases occur in the watershed areas of the parietal and occipital lobes, but PRES has been shown to affect several areas of the brain [2,4,8,9]. The etiology of PRES is not fully understood but thought to be related to vasogenic edema or endothelial dysfunction secondary to a wide array of causative factors. These factors include hypertension, renal failure, eclampsia, preeclampsia, diabetic ketoacidosis, sepsis, cytotoxic drugs, and/or autoimmune disorders such as systemic lupus erythematosus (SLE) [1-11].

The prevalence of PRES in patients with SLE is recorded as much as 0.43% in a case-control study [4]. PRES in SLE affects more women than men (estimated 14:1), with a reported prevalence of 0.7-1.80% patients with SLE [3,4,9]. Although rare among SLE patients, PRES is associated with a high mortality rate [9]. In an analysis of 87 cases, PRES tended to occur in patients with a mean age of 26.3 years and a standard deviation of 8.8 years [4,5]. Risk factors associated with worse outcomes in these patients are white blood cell (WBC) count >9 x 106, urine protein: creatinine ratio >1.00, hemoglobin <10 g/dL, intracranial hemorrhage, and brainstem involvement [3,4,9]. The Systemic Lupus Erythematosus Disease Activity Index (SLEDAI) criteria for lupus was higher (≥6 points) in patients that developed PRES, indicating higher severity of disease at the time of diagnosis [4,12].

Although acutely reversible when the underlying cause is appropriately treated, delayed treatment of PRES in SLE can lead to significant morbidity and mortality. Thus making an early diagnosis of paramount importance [1-11]. While rare, patients with multiple risk factors for PRES should be monitored closely for disease management and prevention. Here we present a case of PRES as an acute manifestation of hypertensive urgency in a 21-year-old female with previously undiagnosed SLE and lupus nephritis.

## Case presentation

**Figure 1:**
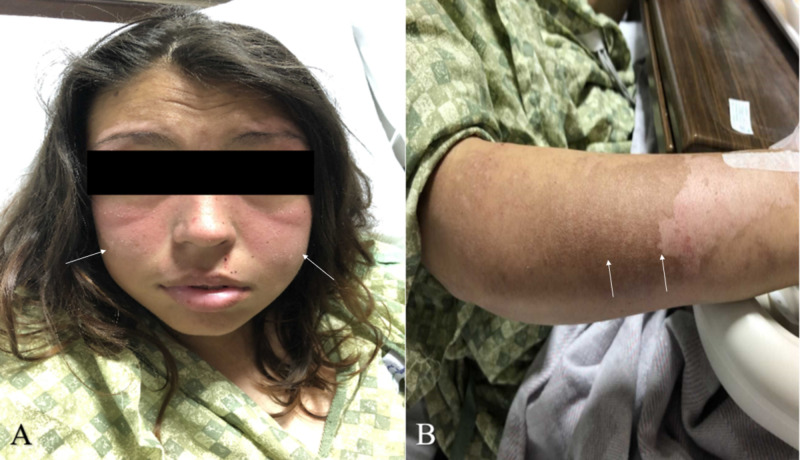
(A) demonstrates characteristic butterfly shaped erythemaous, mildly scaly, macular rash with nasolabial sparing on buccal area is shown (arrows); (B) shows mixed hyperpigmented and hypopigmented scaled skin area on lateral forearm with some erythema consistent with photosensitivity reaction of lupus (arrows)

A 21-year-old female with a past medical history of hypertension and polysubstance abuse presented to our facility with altered mental status, visual disturbances, and new-onset seizures. Prior to arrival, she had developed a rash on her face and arms of two-week duration associated with sun exposure and complained of polyarthralgia (Figure 1).

She was previously on amlodipine and hydrochlorothiazide; however, both medications were recently discontinued. Vital signs on admission included a blood pressure of 200/108 mmHg, heart rate of 95 beats per minute, a respiratory rate of 18 breaths per minute, temperature of 97.7 degrees Fahrenheit, and oxygen saturation of 97% on ambient air. On examination, she had a malar rash, oral ulcers, and a rash covering the dorsal aspects of her forearms. Initial labs were remarkable for normocytic anemia, markedly elevated creatinine, and potassium. Antinuclear antibodies (ANA) and double-stranded deoxyribonuclease (dsDNA) antibodies were both positive. C3 and C4 complement factors were low (Table 1). 

**Table 1:** Laboratory findings ESR: erythrocyte sedimentation rate, CRP: C-reactive protein, ANA: anti-nuclear antibody, dsDNA Ab: double-stranded deoxyribonucleic acid antibody, WBC: white blood cell, CPK: creatine phosphokinase

Lab	Value	Reference range
ESR	80	0-20 mm/hr
CRP	4.0	0- 0.9 mg/dL
ANA	Positive	Positive
dsDNA Ab	>300	0-4.9 IU/mL
Complement C3	<40	90-180 mg/dL
Complement C4	<8.0	14-44 mg/dL
Creatinine	2.40	0.60-1.30 mg/dL
Potassium	6.9	3.5-5.1 mmol/L
Bicarbonate	16	22-34 mmol/L
Hemoglobin	8.9	11.0-15.4 g/dL
WBC Count	14.9	3.7-11.0 thou/mm^3^
CPK	1437	38-234 unit/L

**Figure 2:**
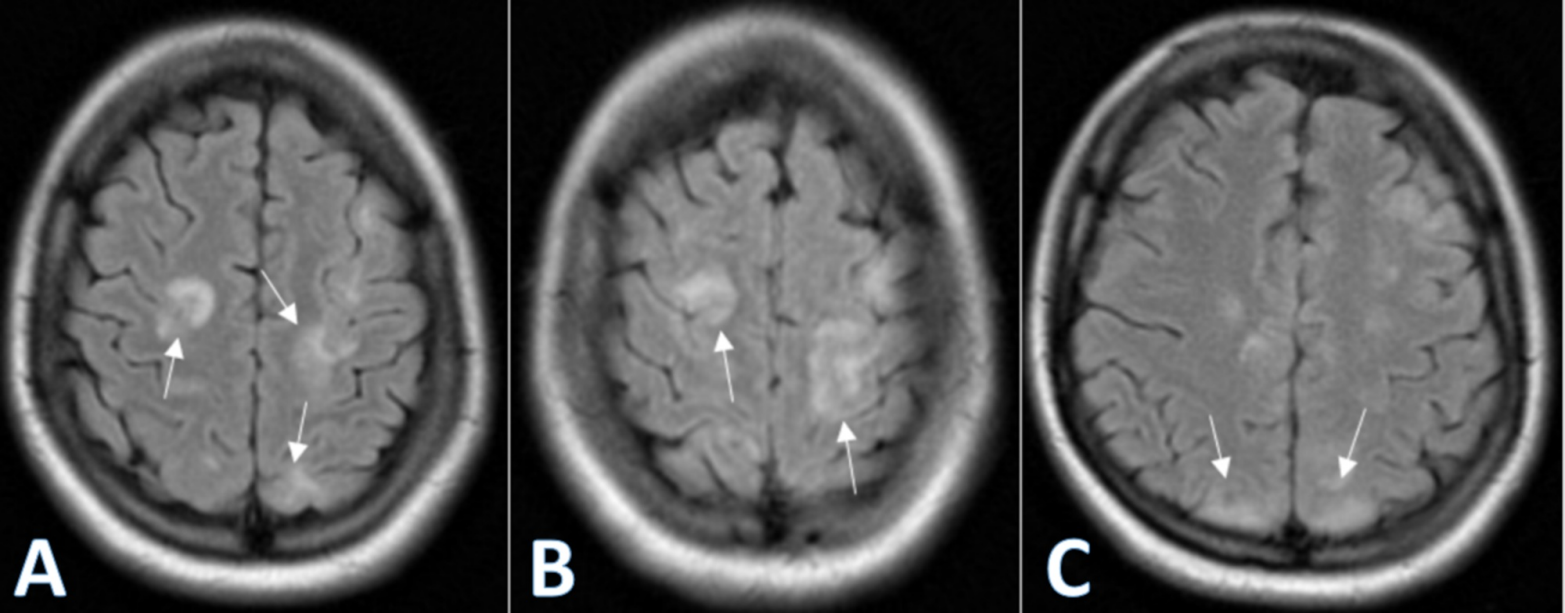
FLAIR imaging of brain MRI demonstrating cortical and subcortical patchy edema with increased signal intensity: (A,B) demonstrate patchy involvement of the parietal, temporal, and occipital lobes (arrows); (C) demonstrates bilateral occipital lobe involvement (arrows) FLAIR: fluid-attenuated inversion recovery

Urinalysis revealed proteinuria, with albumin-to-creatinine ratio (ACR) ≥3.5 grams/day consistent with nephrotic range proteinuria. Magnetic Resonance Imaging (MRI) of the brain was performed and showed patchy cortical and subcortical edema in posterior lobes suggestive of PRES (Figure 2).

She was given intravenous (IV) lorazepam and labetalol in the emergency department, which alleviated seizure activity and normalized blood pressure. The patient met diagnostic criteria for SLE, and was subsequently started on methylprednisolone pulse therapy, given concerns for active proliferative lupus nephritis, with significant improvement in arthralgia and kidney function. Kidney biopsy later confirmed stage V membranous lupus nephritis. The patient was ultimately transitioned to oral prednisone taper, started on mycophenolate mofetil, and provided pneumocystis pneumonia (PCP) prophylaxis with atovaquone. 

## Discussion

Although the exact disease mechanism underlying PRES in SLE is poorly understood, the most widely accepted PRES theory involves hypertension (mean arterial pressure of approximately 150-160 mmHg), disrupting of brain autoregulation and causing vasogenic edema. In addition, renal dysfunction, hypoalbuminemia, and thrombocytopenia are independent risk factors of PRES, and potentially linked to the disease [2]. Kalaiselvan et al. argue that the progression of PRES is caused by endothelial damage, exacerbated by vasoconstriction, and autoimmune activation [13]. All these conditions may be coinciding, creating a summative or multiplicative effect on the course of the disease.

CT imaging is often utilized first in the emergency department to rule out cerebral hemorrhage or increased intracranial pressure. While CT scans may show abnormalities consistent with PRES, these changes are best visualized on MRI [14]. Once hemorrhage is ruled out, lumbar puncture (LP) may be performed if there is suspicion of meningitis, encephalitis, or malignancy. Cerebrospinal fluid (CSF) in PRES often shows modestly elevated protein with no pleocytosis [15]. Fluid-attenuated inversion recovery (FLAIR) is the imaging modality of choice to diagnose PRES and may display findings of white matter edema in the watershed areas of the posterior cerebrum. PRES has been shown to affect the posterior regions of the parietal and occipital lobes most commonly but has also been shown to affect the frontal and temporal regions with relative frequency [1,2,4-10,13].

PRES is seen more frequently in women, with a median age of onset of 31.5 to 48 years, and range of 9-82 years [12,16]. Common triggers of PRES include eclampsia, lupus nephritis, drug abuse (e.g., cocaine), autoimmune disease, kidney disease, and hypertension. Among the 113 patients with PRES syndrome in the Fugate et al. study, the most common presenting symptoms were seizure (74%), followed by encephalopathy (28%), headaches (26%), and visual disturbances (20%) [16]. The patient described in this case report had all the symptoms described above in the context of lupus nephritis, autoimmunity, and essential hypertension.

It is essential to consider an extensive differential diagnosis when a patient presents with seizures and encephalopathy in SLE. Neuropsychiatric symptoms in SLE are relatively common, and the American College of Rheumatology (ACR) criteria delineate guidelines for appropriate work-up and treatment for each condition [17]. PRES is not included in ACR nomenclature and therefore is a diagnosis of exclusion with supportive findings. Differential diagnoses include but are not limited to a primary seizure disorder, lupus cerebritis, meningitis, cerebral ischemia, and many more [3]. Lupus cerebritis is a particularly important differential diagnosis to PRES, as it presents with similar symptoms, antibodies in the CSF, and nonspecific changes on brain MRI. Imaging in PRES is distinct from lupus cerebritis; however, the vasogenic edema tends to affect specific foci of the brain and may resolve within several days of appropriate treatment. Diffusion-weighted imaging (DWI) is useful in differentiating PRES from cerebral ischemia, as DWI shows hyperintense lesions in ischemic damage (cytotoxic edema) and hypodense lesions in PRES (vasogenic edema) [6].

## Conclusions

Active SLE, renal failure, and uncontrolled hypertension are known risk factors for the development of PRES. PRES can mimic neuropsychiatric SLE or other pathologies and is therefore under-recognized or frequently misdiagnosed. This case highlights the importance of considering PRES as a differential diagnosis in a patient with seizure and altered mental status in the context of SLE. This patient’s presentation to the hospital may have been prevented with appropriate diagnosis and medical management of her chronic conditions. Early diagnosis of PRES is key to successful management.
